# Monitoring the Increase in the U.S. Smoking Cessation Rate and Its Implication for Future Smoking Prevalence

**DOI:** 10.1093/ntr/ntac115

**Published:** 2022-04-29

**Authors:** David Méndez, Thuy T T Le, Kenneth E Warner

**Affiliations:** Department of Health Management and Policy, School of Public Health, University of Michigan, Ann Arbor, MI 48109, USA; Department of Health Management and Policy, School of Public Health, University of Michigan, Ann Arbor, MI 48109, USA; Department of Health Management and Policy, School of Public Health, University of Michigan, Ann Arbor, MI 48109, USA

## Abstract

**Introduction:**

We calculate the U.S. adult smoking cessation rate for 2014–2019, compare it to the historical trend, and estimate the implication for future smoking prevalence.

**Methods:**

We repeated an earlier analysis, which examined the cessation rate from 1990 to 2014, extending the period to 2019. Employing National Health Interview Survey (NHIS) and National Survey on Drug Use and Health (NSDUH) data, we estimated the adult cessation rate in 6-year intervals, using weighted nonlinear least squares. We then employed a meta-regression model to test whether the cessation rate has increased beyond expectation. We used cessation rate estimates and smoking initiation rate estimates to project smoking prevalence in 2030 and eventual steady-state prevalence.

**Results:**

The annual cessation rate increased 29% using NHIS data (from 4.2% in 2008–2013 to 5.4% in 2014–2019) and 33% with NSDUH data (4.2%–5.6%). The cessation rate increase accounts for 60% of a smoking prevalence decline in the most recent period exceeding the 1990–2013 predicted trend. The remaining 40% owes to declining smoking initiation. With current initiation and cessation rates, smoking prevalence should fall to 8.3% in 2030 and eventually reach a steady state of 3.53%.

**Conclusions:**

The smoking cessation rate continued to increase during 2014–2019. NHIS and NSDUH results are practically identical. The larger share (60%) of the smoking prevalence decrease, beyond expectation, attributable to the increased cessation rate is encouraging since the positive health effects of cessation occur much sooner than those derived from declining initiation.

**Implications:**

The smoking cessation rate in the United States continues to increase, accelerating the decline in smoking prevalence. This increase suggests that the Healthy People 2030 goal of 5% adult smoking prevalence, while ambitious, is attainable. Our findings can be used in simulation and statistical models that aim to predict future prevalence and population health effects due to smoking under various scenarios.

## Introduction

In a 2017 article,^1^ Mendez et al. showed that the U.S. adult smoking cessation rate increased almost steadily during 1990–2014. The authors analyzed data from the National Health Interview Survey (NHIS) for 1990–2014 and the National Survey on Drug Use and Health (NSDUH) for 2002–2014, obtaining nearly identical results from both surveys for the comparable years. Because of survey design changes in 2002 that prevented comparability with previous years,^2^ they excluded NSDUH data prior to 2002 from the analysis. Based on the NHIS data, the authors found that the annual adult cessation rate increased from 2.4% in 1990–1995 to 4.5% in 2008–2014. (The authors evaluated the cessation rate in groups of 6 years—7 in the case of 2008–2014—to conform to the assumptions of their model.^1^) Based on the NSDUH data, the cessation rate increased from 3.2% in 2002–2007 to 4.2% in 2008–2014. The findings showed a highly statistically significant positive trend for the cessation rate over the periods considered for both surveys (*p* = 1.57 × 10^−6^).

Using the results based on the NHIS data, the authors reported the eventual attainment of a steady-state smoking prevalence of 6.5% if the smoking initiation and cessation rates observed in 2008–2014 continued indefinitely. In that work, the authors defined the cessation rate in a particular year as the proportion of smokers who quit permanently (net of relapses) in that year. The initiation rate was defined as the number of 18-year-old smokers expressed as a proportion of the entire adult population. NHIS provides smoking prevalence estimates for 18–24 year olds and the authors considered this prevalence to represent the initiation rate for 18 year olds. To calculate the number of 18-year-old smokers, the authors multiplied this prevalence by the number of 18 year olds from the U.S. Census, which also supplied the size of the entire adult population.^3^

In the present study, we repeat the analysis presented in Mendez et al.,^1^ extending the evaluation period to 2019. The aim of the paper is threefold. First, we want to estimate changes in the adult cessation rate in the most recent 6-year period. By doing so, we are able to not only assess the current status of this important indicator of the smoking epidemic but also inform forecasting and simulation models attempting to predict the likely path of adult population smoking prevalence and its future health consequences. Second, we want to assess whether the cessation rate value obtained for the most recent period is significantly above or below the expectation derived from previous periods’ data. That is, has the long-term growth trend in the cessation rate remained the same, sped up, or slowed down during the most recent 6-year period? Third, we want to re-evaluate the feasibility of the Healthy People goal of achieving 5% adult smoking prevalence by 2030. In the 2017 article,^1^ Mendez et al. concluded that achieving that target was not feasible. Our new analysis could support or amend that conclusion. If the latter, the results would be welcome news to the public health community and would serve as a source of renewed optimism and enthusiasm about devoting effort to future tobacco control interventions.

## Methods

We followed the same analysis procedure as in Mendez et al.^1^ and estimated the adult cessation rate by 6-year periods, from 1990 to 2019, employing the following model:


$$\begin{array}{l} \pi (t)=\left(\pi \left(0\right)-\left(\frac{\lambda }{\theta +\mu }\right)\right)\times e^{-\left(\theta +\mu \right)\times t}+\left(\frac{\lambda }{\theta +\mu }\right) \end{array}$$ (1)

where π(t) is adult smoking prevalence in year *t*, and λ, θ, and μ correspond to the 6-year estimation period initiation, cessation, and smoker mortality rates, respectively, defined as in the 2017 study. The model has been used in other studies both in its current compact form^1,3^ and in a more disaggregated formulation.^4–7^

As in the 2017 study, we focus on permanent quit rates (net of relapses) and use the study’s definition for the initiation rate: the number of 18-year-old smokers expressed as a proportion of the entire adult population. Since the initiation rate is often expressed as the smoking prevalence of 18-year-olds, for clarity Supplementary Table A1 translates our current definition into the more familiar smoking prevalence of 18-year-olds. A detailed description and derivation of the model are presented in Supplementary Appendix, pp. 4–5.

To estimate the model, we used data from the NHIS^4^ and NSDUH^5^ surveys. NHIS and NSDUH are nationally representative household surveys of the civilian non-institutionalized U.S. population and are main sources of data on smoking. Starting in 1990, NHIS smoking data were collected on an annual basis, with the exception of the year 1996. Current smoking is defined in NHIS as having smoked at least 100 cigarettes in one’s lifetime, and smoking “now” for 1990 and 1991, or smoking “every day” or “some days” for the years 1992–present. The definition introduced in 1992 was reported to have increased the estimate of smoking prevalence by approximately 1 percentage point^8^ so we increased smoking prevalence estimates in 1990 and 1991 by 1 point to adjust for the change in definition. The NSDUH standard definition for current smoking is any cigarette use in the past month, where cigarette use is defined as smoking “part or all of a cigarette”. Because of survey design changes in 2002 that prevent comparability with previous years,^2^ we excluded NSDUH data prior to 2002 from our analysis. Prevalence estimates for NSDUH are consistently 4.5–6 points higher than those for NHIS and the initiation rate estimates are twice as large. This is due to the different definitions for current smoker between the two surveys.^1,9,10^ Overall smoker death rates were computed using age-gender-and-year-specific smoker death rates obtained from the Cancer Intervention and Surveillance Modeling Network (CISNET).^11^ The CISNET rates were weighted by the corresponding population size to calculate the overall annual smoker death rate.

To carry out the estimation, we included the following updates to the 2017 study:

a) We added a new estimation period for the cessation rate, 2014–2019. In the case of NHIS, however, while 2019 prevalence data are available, we did not use them due to changes in the 2019 NHIS survey that rendered that year’s observations incompatible with previous years’ data. As such, when using NHIS we treated 2019 as missing data for the period 2014–2019. NHIS adjusted the weighting method in 2019 to deal with large decreases in response rates. These weighting and other design methodology changes can affect comparisons of weighted survey estimates over time.^12^
b) We moved the 2014 data to the last estimation period to include 6 years in each period. Consequently, the next to the last estimation period changed from 2008-2014 in the previous analysis^1^ to 2008–2013.
c) We corrected an input error in the initiation rate for the period 2002-2007 in the earlier paper.^1^ As a result, the initiation rate in that period changed from 0.41% to 0.45%.

With the updated dataset, we used weighted least squares (WLS)^13^ and the model shown in expression (1) to estimate the smoking cessation rate for each of the five analysis periods (1990–1995, 1996–2001, 2002–2007, 2008–2013, 2014–2019).

Finally, we performed a meta-regression^14^ using the cessation rate estimates for each survey and time-interval through 2014–2019 to estimate the model, and tested a significant departure from trend for the 2014–2019 period. To perform the meta-regression, we used a linear mixed model with Gaussian random effects:


$$\begin{array}{l} \;\overset{}{\hat{\mu }}\;(t)=\;\beta_{0}+\beta_{1}\times I_{NSDUH}+\beta_{2}\times t\;+\;\beta_{3}\times I_{2014-2019} \end{array}$$ (2)

where $I_{NSDUH}$ is an indicator of the data source (1 = NSDUH, 0 = NHIS), $I_{2014-2019}$ is an indicator of the 2014–2019 period (1 = 2014–2019 datapoint, 0 = otherwise), and *t* is a variable that indexes sequential periods. The model was fit by maximum likelihood.

The data used in this study and the complete estimation results are presented in Supplementary Tables A1–A3).

All the statistical analyses were performed using the R package version 4.0.3. For a detailed exposition of the methods, we refer the readers to Supplementary Appendix.

## Results

Table 1 shows the results of our analysis. By moving the 2014 data to the last period and correcting the initiation rate value for the period 2002–2007, our estimates of the cessation rates using NHIS data differ from the earlier ones^1^ as follows. The cessation rate estimate for the period 2002–2007 changed from 3.3% to 3.5%, and the estimate for the 2008–2013 (formerly 2008–2014) period adjusted from 4.5% to 4.2%. The estimates obtained from the NSDUH data for 2002–2007 and 2008–2013 (formerly 2008–2014) did not change from their previously estimated values of 3.2% and 4.2%, respectively.

The results of the meta-regression through the 2014–2019 period are similar to those of the previous study. The estimated trend coefficient indicates an increase in the cessation rate of 0.50 percentage points per year (95% CI = 0.20%, 0.80%) and remains statistically significant (*p* = 1.02 × 10^−3^). The 2014–2019 variable is significant at the 5% level (*p* = 1.82 × 10^−2^), indicating a 1.07 (95% CI = 0.18, 1.96) percentage point increase above trend in the 2014–2019 cessation rate. As in the previous study,^1^ the survey-specific variable is not significant at the 5% level (*p* = 3.05 × 10^−1^).

We ran another meta-regression excluding the nonsignificant indicator for the survey-specific variable ($I_{NSDUH})$ and obtained almost identical results. In particular, the 2014–2019 indicator was again significant at the 5% level (*p* = 1.60 × 10^−2^) with an estimated value of a 1.09-percentage-point increase above trend (95% CI = 0.20, 1.98). The entire meta-regression results are shown in Supplementary Tables A4 and A5.

Following our previous analysis,^1^ we updated the estimate for the eventual steady-state prevalence (SSP) using the initiation and cessation rates estimated for the last analysis period (2014–2019) based on the NHIS data, obtaining SSP = 3.53%. (See computations in Supplementary Appendix, p. 7). The steady state is the value at which smoking prevalence will stabilize and remain constant if the initiation and cessation rates do not change from their current levels. In our case, it measures the long-term effects of the conditions that currently exist. Using the same parameter values, our model estimates that U.S. adult smoking prevalence in 2030 will be 8.3% (95% CI = 4.6%, 16.8%).

Additionally, we estimated the portion of the decline in the annual NHIS smoking prevalence between 2013 and 2019 explained by the changes in initiation and cessation rates over the same period. To do this, we calculated what the prevalence in 2019 would have been (14.9%) if the cessation and initiation rates had remained constant at their 2008–2013 levels and subtracted from it the model-projected prevalence in 2019 (13.3%) using 2014–2019 initiation and cessation rates, obtaining a difference of 1.6 percentage points. Our model shows that the increase in cessation between 2008–2013 and 2014–2019 is responsible for 60% (0.96 percentage points) of the extra fall in prevalence, and the remaining 40% (0.64 percentage points) can be attributed to the decrease in the initiation rate between those periods. (See the computations in the Supplementary Appendix, pp. 8–12.)

Finally, we computed the proportion of the total fall in smoking prevalence from 2013 to 2019 attributable to the changes in initiation and cessation rates that occurred during 2014–2019. First, we subtracted the estimated NHIS smoking prevalence in 2019 (13.28%) obtained with the most current parameters for the initiation (0.22%) and cessation (5.4%) rates, from the estimated NHIS smoking prevalence in 2013 (17.71%). We used estimated, rather than the reported value of smoking prevalence in 2013, to avoid observation noise. We do not have an NHIS smoking prevalence reported value for 2019. The estimated total smoking prevalence drop from 2013 to 2019 is then 17.71% –13.28% = 4.4 percentage points. Since we already found that the total prevalence drop attributable to the changes in initiation and cessation rates from their 2007–2013 values to their 2014–2019 values is 1.6 percentage points, it follows that these recent changes are responsible for 1.6/4.4 = 36% of the total prevalence drop. The remaining 64% is then due to causes that affected the initiation and cessation rates before 2014.

**Table 1.** Estimated Smoking Cessation Rate by Period and Survey, 1990–2019

Period	NHIS (%)	NSDUH (%)
1990–1995	2.4 (1.5, 3.3)	
1997–2001	3.4 (3.1, 3.7)	
2002–2007	3.5 (2.7, 4.3)	3.2 (2.6, 3.8)
2008–2013	4.2 (3.5, 4.9)	4.2 (3.6, 4.8)
2014–2019	5.4 (3.1, 7.6)	5.6 (4.9, 6.3)

Figures in parentheses represent 95% CIs.

## Discussion

The smoking cessation rate in the United States continued to increase during 2014–2019. Our results show a 29% increase from the 2008–2013 value using NHIS data (4.2%–5.4%) and a 33% increase using the NSDUH data (4.2%–5.6%). The overall results from both surveys continue to be practically identical, which lends validity to the study. Going forward, using the NHIS estimates of initiation and cessation rates for 2014–2019, we project that smoking prevalence in 2030 will be 8.3% (95% CI = 4.6%, 16.8%). Note that using the current estimates of initiation and cessation rates is likely conservative, given that continuing improvements in both are likely to occur. This suggests that the CDC Healthy People goal of 5% adult smoking prevalence in 2030,^15^ while still ambitious, is attainable.

The sustained growth in the cessation rate, and especially its acceleration in the most recent years, is very encouraging. We do not know what accounts for this continuing increase. It could be that evidence-based tobacco control measures in play for many years now, such as taxation, smoke-free air laws, and cessation treatment, are still driving up the cessation rate. It is also possible that, as more people quit, smokers have a reduced social network to help them sustain their habit, which leads to an accelerated quit rate.^16^ The use of new noncombusted nicotine delivery products, such as e-cigarettes, which offer smokers an alternative source of nicotine, may also be helping to increase the smoking cessation rate.^17,18^ The most likely explanation may well be a combination of the above.

The ultimate effect of an increase in the cessation rate is a reduction in smoking prevalence, driven not only by cessation, but by changes in the smoking initiation rate as well. We have witnessed a substantial decrease in the initiation rate over the last 6 years (from 19.7% in 2008–2013 to 12.2% in 2014–2019, according to the NHIS data [Supplementary Tables A1 and A2]). However, the recent increase in the cessation rate is responsible for the majority of the excess reduction in smoking prevalence over the same period (60% cessation vs. 40% initiation).

Finally, we would like to highlight the long memory inherent in the processes that drive smoking prevalence. The positive changes in the initiation and cessation rates during the last 6 years have certainly contributed to the drop in prevalence during this period. However, they are responsible only for 1.6 percentage points (36%) of the total 4.4-percentage-point prevalence decline. The rest (64%) can be attributed to the cumulative effect of past changes in smoking initiation and cessation. A better measure of the total impact of current initiation and cessation rates is the impressive drop in the steady-state prevalence (3.53% in the present study vs. 6.5% in the earlier study^1^), a measure of the lowest prevalence we can expect in the future, given current initiation and cessation rates. Of course, if the trends of increasing cessation and decreasing initiation rates persist, steady-state prevalence will fall further, as may prevalence in 2030.

Our study is not without limitations. As in the 2017 study,^1^ it is possible that there is under-reporting of smoking behavior in both surveys due to the respondents’ perceptions of the social acceptability of smoking. However, we believe that such under-reporting has not grown over the timespan of our analysis, and thus will not introduce a temporal bias into our results. Liber and Warner^19^ examined this question and concluded that under-reporting has not grown over time from 1965 to 2015 in either survey.

Another potential limitation could be associated with the length of our fixed estimation intervals. It is possible that 6-year periods may obscure shorter-term differences in the cessation rate. However, there is a good reason for choosing the 6-year intervals. The prevalence model we use for estimation (derived in our previous paper) assumes fixed initiation and cessation rates over a period of time. As such, we are calculating an average cessation rate over the period. Having at least six data points per period is necessary to have enough power to detect statistically significant results, avoid overfitting, and smooth out observation errors in the initiation rate. Shorter intervals would produce inconsistent estimates (from period to period) that reflect more the noise in the observations than the information in the series.

Remarkable decreases in initiation bode well for the future of tobacco control. Still, the large and continuing increase in the cessation rate is the variable that most immediately addresses the enormity of the toll of disease and death that smoking continues to exact from the health of the American people. The finding that smoking cessation in the United States is not only increasing, but that it is doing so at an accelerated rate, promises a brighter future for the battle against Public Health Enemy Number 1.

## Supplementary Material

A Contributorship Form detailing each author’s specific involvement with this content, as well as any supplementary data, are available online at https://academic.oup.com/ntr.

ntac115_suppl_Supplementary_Appendix

ntac115_suppl_Supplementary_Taxonomy-Form

## Data Availability

All the data used in this work are listed in Supplementary Appendix.
