# Supplementary material for: Monitoring the Increase in the U.S. Smoking Cessation Rate and Its Implication for Future Smoking Prevalence
Source: Nicotine Tob Res. 2022 Apr 29;24(11):1727–31. doi: 10.1093/ntr/ntac115 (PMC9596994; doi:10.1093/ntr/ntac115)
Supplement: ntac115_suppl_Supplementary_Appendix [file ntac115_suppl_supplementary_appendix.pdf]

APPENDIX TO **"MONITORING THE TREND OF THE U.S. SMOKING CESSATION RATE AND ITS IMPLICATION FOR FUTURE SMOKING PREVALENCE"**

David Méndez, Ph.D.
Department of Health Management and Policy
School of Public Health, University of Michigan
1415 Washington Heights, Ann Arbor, MI 48109

Thuy T.T. Le, Ph.D.
Department of Health Management and Policy
School of Public Health, University of Michigan
1415 Washington Heights, Ann Arbor, MI 48109

Kenneth E. Warner, Ph.D.
Department of Health Management and Policy
School of Public Health, University of Michigan
1415 Washington Heights, Ann Arbor, MI 48109

Table A1. Adult Smoking Initiation Rate as a Percentage of the Adult and the 18-year-old Populations

	**NHIS**		**NSDUH**	
**Year**	**Init Rate (% of adult population)**	**Init Rate (% of 18-year-olds)**	**Init Rate (% of adult population)**	**Init Rate (% of 18-year-olds)**
1990-1995	0.47	25.7		
1996-2001	0.55	27.6		
2002-2007	0.45	24.4	0.83	39.3
2008-2013	0.35	19.7	0.69	33.5
2014-2019	0.22	12.2	0.43	23.1

Table A2. Model Input Data Used to Estimate Smoking Cessation Rates, 1990-2019

		NHIS			NSDUH		
Year	Death Rate (μ) (%)	Smoking Prevalence (%)	(SE) (%)	Init Rate (λ) (%)	Smoking Prevalence (%)	(SE) (%)	Init Rate (λ) (%)
1990		26.5	(0.30)				
1991		26.6	(0.20)				
1992		26.5	(0.26)				
1993	0.94	25.0	(0.36)	0.47			
1994		25.5	(0.36)				
1995		24.7	(0.41)				
1996							
1997		24.7	(0.31)				
1998		24.1	(0.31)				
1999	0.88	23.5	(0.31)	0.55			
2000		23.3	(0.26)				
2001		22.8	(0.26)				
2002		22.5	(0.31)		27.5	(0.37)	
2003		21.6	(0.31)		26.9	(0.38)	
2004		20.9	(0.31)		26.6	(0.32)	
2005	0.84	20.9	(0.31)	0.41	26.8	(0.41)	0.83
2006		20.8	(0.36)		26.7	(0.36)	
2007		19.8	(0.41)		25.8	(0.37)	
2008		20.6	(0.41)		25.5	(0.44)	
2009		20.6	(0.36)		25.2	(0.37)	
2010		19.3	(0.31)		24.6	(0.38)	
2011	0.89	19.0	(0.31)	0.35	23.6	(0.40)	0.69
2012		18.1	(0.31)		23.8	(0.37)	
2013		17.8	(0.31)		22.8	(0.33)	
2014		16.8	(0.33)		22.7	(0.28)	
2015		15.1	(0.28)		21.0	(0.31)	
2016		15.5	(0.33)		20.7	(0.33)	
2017	0.89	14.0	(0.31)	0.22	19.4	(0.25)	0.43
2018		13.7	(0.31)		18.9	(0.29)	
2019					18.2	(0.25)	

Init Rate = Smoking prevalence among 18–24-year-olds as a proportion of the adult population; NHIS = National Health Interview Survey; NSDUH = National Survey on Drug Use and Health; SE = standard error.

Table A3. Estimated Cessation Rates by Period and Data Source

Period / Data Source	Parameter	Estimate	Std Error	Statistic	p-Value
1990-1995 NHIS	pi0	0.269	0.003	89.773	0.000
1990-1995 NHIS	theta	**0.024**	0.005	5.124	0.007
1996-2001 NHIS	pi0	0.287	0.004	66.337	0.000
1996-2001 NHIS	theta	**0.034**	0.001	22.817	0.000
2002-2007 NHIS	pi0	0.302	0.019	15.828	0.000
2002-2007 NHIS	theta	**0.035**	0.004	8.803	0.001
2008-2013 NHIS	pi0	0.418	0.036	11.736	0.000
2008-2013 NHIS	theta	**0.042**	0.004	11.359	0.000
2014-2019 NHIS	pi0	0.613	0.197	3.112	0.053
2014-2019 NHIS	theta	**0.054**	0.011	4.672	0.019
2002-2007 NSDUH	pi0	0.318	0.017	18.786	0.000
2002-2007 NSDUH	theta	**0.032**	0.003	10.785	0.000
2008-2013 NSDUH	pi0	0.438	0.034	12.876	0.000
2008-2013 NSDUH	theta	**0.042**	0.003	13.742	0.000
2014-2019 NSDUH	pi0	0.814	0.082	9.866	0.001
2014-2019 NSDUH	theta	**0.056**	0.003	16.555	0.000

Theta = Cessation Rate; pi0 = Prevalence at time = 0 (Estimated for each period-data-source model)

Table A4. Meta-regression Results to Test for Changes in Smoking Cessation Rates, 1990–2019 (with Survey Indicator Variable)

Variable	Coefficient Estimate (%)	p-value
t	0.50 (0.20, 0.80)	1.02×10^{-3}
I_{NSDUH}	-0.31 (-0.90, 0.28)	3.05×10^{-1}
$I_{2014\text{-}2019}$	1.07 (0.18, 1.96)	1.82×10^{-2}

Figures in parentheses represent 95% confidence intervals

Table A5. Meta-regression Results to Test for Changes in Smoking Cessation Rates, 1990–2019 (w/o Survey Indicator Variable)

Variable	Coefficient Estimate (%)	p-value
t	0.41 (0.17, 0.65)	8.64×10^{-4}
$I_{2014\text{-}2019}$	1.09 (0.20, 1.98)	1.60×10^{-2}

Figures in parentheses represent 95% confidence intervals

Estimation Model Formulation
(From Mendez et. al. 2017 [1])

To estimate the overall adult smoking cessation rate, we employ a stock-and-flow approach to describe smoking prevalence as used in a previous dynamic model of population smoking [2, 3]. The basic approach can be described by the following ordinary differential equation:

$$\frac{dS(t)}{dt} = \Big(I(t) - \big(\mu(t) + \theta(t)\big) \times S(t)\Big) \tag{1}$$

where $S(t)$ represents the number of adult smokers in the population at time t, $I(t)$ is the number of new adult smokers per year at time t, and $\theta(t)$ and $\mu(t)$ stand for the cessation and smoker death rates at time t, respectively.

Expression (1) states that the rate of change in the number of smokers depends on the difference between the rate at which new smokers are generated and the speed at which existing smokers leave the smoking pool because of cessation or death. This formulation does not show an explicit link between the rate of new smokers and the size of the pool of smokers, thus treating initiation as an exogenous variable. On the other hand, expression (1) does imply that the smoker exit rate depends on the number of smokers in the population. As such, the cessation rate can be interpreted as the probability that a smoker quits. Because we do not observe actual quitting, but smoking prevalence at different periods, we can only infer the cessation rate net of relapses, which we take as a proxy for permanent quitting. In this work, we consider only permanent quits.

Holding the rate parameters constant over a specified period of time, expression (1) can be solved as:

$$S(t) = \left(S(0) - \left(\frac{I}{\theta + \mu}\right)\right) \times e^{-(\theta+\mu)\times t} + \left(\frac{I}{\theta + \mu}\right) \tag{2}$$

where e is the base of the natural logarithms.

Let $P(t)$ be the size of the adult population at time *t*. Assuming a constant population P over each time period of analysis, we can express adult smoking prevalence $\pi(t)$ as:

$$\pi(t) = \frac{S(t)}{P} = \left(\frac{S(0)}{P} - \frac{1}{P} \times \left(\frac{I}{\theta + \mu}\right)\right) \times e^{-(\theta+\mu)\times t} + \frac{1}{P} \times \left(\frac{I}{\theta + \mu}\right) \tag{3}$$

Let $\lambda = \frac{I}{P}$ be the smoking initiation rate expressed as the proportion of the adult population that starts smoking every year. Then expression (3) becomes:

$$\pi(t) = \left(\pi(0) - \left(\frac{\lambda}{\theta + \mu}\right)\right) \times e^{-(\theta+\mu)\times t} + \left(\frac{\lambda}{\theta + \mu}\right) \tag{4}$$

We use expression (4) to estimate values for the cessation rate (θ), controlling for the initiation rate (λ). To conduct the estimation, we used adult smoking prevalence data from the National Health Interview Survey (NHIS) and the National Survey on Drug Use and Health (NSDUH). We use smoking prevalence among 18-24 year-olds as a proportion of the entire adult population as a proxy for the smoking initiation rate, thus assuming that little or no initiation occurs after age 24, consistent with existing data [4]. Notice that, while we restrict prevalence to ages 18-24 to estimate initiation rates (λ), we use adult smoking prevalence for all ages to conduct the estimation of the cessation rate; as such, our cessation rate estimates represent an average across all adult ages.

Figure A1. Estimated vs. Observed U.S. Adult Smoking Prevalence

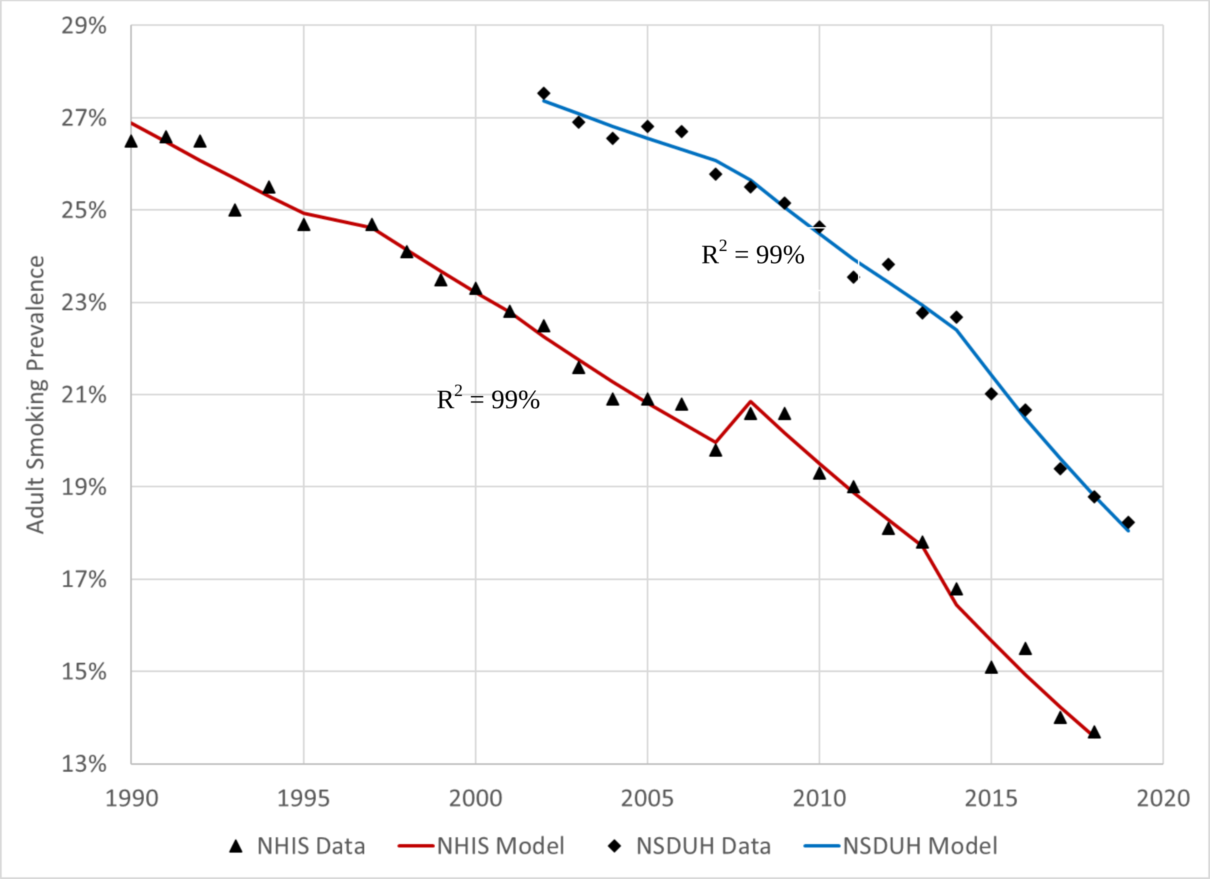

Computation of the U.S. Steady State Smoking Prevalence

In our model, the steady-state prevalence (SSP) represents the level that the US prevalence will ultimately approach if current conditions remain stable (i.e., same initiation and cessation rates.) It is an indicator of the long-term effect of the current levels of initiation and cessation. We compute the SSP in the following way:

From expression (1) in the main text, the steady-state value for the U.S. smoking prevalence can be computed as:

$$SSP = \lim_{t \to \infty} \pi(t) = \lim_{t \to \infty} \left(\pi(0) - \left(\frac{\lambda}{\theta + \mu} \right) \right) \times e^{-(\theta+\mu)\times t} + \left(\frac{\lambda}{\theta + \mu} \right) = \left(\frac{\lambda}{\theta + \mu} \right)$$

Since the first term of this expression approaches to zero as *t* goes to infinity,

$$SSP = 0 + \left(\frac{\lambda}{\theta + \mu} \right) = \left(\frac{\lambda}{\theta + \mu} \right)$$

Using the most current cessation rate estimate from the NHIS data, we obtain:

$$SSP = \left(\frac{\lambda}{\theta + \mu} \right) = \left(\frac{0.22\%}{0.89\% + 5.35\%} \right) = 3.53\%$$

Computation of the Relative Contributions of the Changes in Initiation and Cessation during 2014-2019 to the Excess Drop in Smoking Prevalence during the Same Period.

We will calculate these proportions with the NHIS results. First, we use expression (1) in the text to estimate smoking prevalence in 2013 and 2019, obtaining:

$\hat{\pi}_{2008-2013}(2013)$ =17.71% (Estimated prevalence in 2013 using 2008-2013 parameters)

$\hat{\pi}_{2014-2019}(2019)$ =13.28% (Estimated prevalence in 2019 using 2014-2019 parameters)

So, the overall estimated drop in smoking prevalence from 2013 to 2019 is 17.71% - 13.28% = 4.43 percentage points.

If the initiation and cessation rates over 2014-2019 had remained at their 2008-2013 values, smoking prevalence in 2019 would have been:

$\hat{\pi}_{2008-2013}(2019)$ =14.85%

Thus, the prevalence drop that can be attributable to changes in initiation and cessation over 2014-2019 is (14.85% - 13.28% =) 1.57 percentage points, or 35% of the total drop in prevalence during that period (4.43 percentage points.)

We can further examine the relative contribution of the changes in initiation and cessation during 2014-2019 to the 1.57 percentage points drop:

Again, applying expression (1), if the initiation rate during 2014-2019 had decreased to 0.22%, but the cessation rate had remained at its 2008-2013 value (4.2%), prevalence in 2019 would have been

$$\hat{\pi}_{\substack{init\ 2014-2019 \\ cess\ 2008-2013}}(2019) = 14.21\%$$

and, if the cessation rate had increased to 5.4%, while the initiation rate had remained at 0.35%, the 2019 smoking prevalence would have been

$$\hat{\pi}_{\substack{init\ 2008-2013 \\ cess\ 2014-2019}}(2019) = 13.90\%$$

Therefore, the recent decrease in the initiation rate is responsible for (14.85 – 14.21 =) 0.64 percentage points drop in prevalence (or 40% of the 1.57 percentage points drop) while the increase in the cessation rate during 2014-2019 accounts for (14.85 – 13.90) = 0.95 percentage points drop (or 60% of the 1.57 prevalence drop).

The following tables and figures illustrate the analysis. All prevalence estimates were obtained by applying Expression 4, described earlier:

$$\pi(t) = \left(\pi(0) - \left(\frac{\lambda}{\theta + \mu}\right)\right) \times e^{-(\theta+\mu)\times t} + \left(\frac{\lambda}{\theta + \mu}\right)$$

Table A6. Estimated smoking prevalence by 2013 and 2019.

Target Year	Base Year (time 0)	Prevalence at time 0	Initiation rate period	Initiation rate value	Cessation rate period	Cessation rate value	Mortality rate	Estimated prevalence in target year
2013	1990	41.80%	2008-2013	0.35%	2008-2013	4.20%	0.89%	17.71%
2019	2013	17.71%	2008-2013	0.35%	2008-2013	4.20%	0.89%	14.85%
2019	2013	17.71%	2014-2019	0.22%	2014-2019	5.40%	0.89%	13.28%
2019	2013	17.71%	2014-2019	0.22%	2008-2013	4.20%	0.89%	14.21%
2019	2013	17.71%	2008-2013	0.35%	2014-2019	5.40%	0.89%	13.90%

Table A7. Estimated smoking prevalence 2014-2019 under different values of initiation and cessation rates.

Year	2007-2013 Initiation and Cessation Rates	2014-2019 Initiation and Cessation Rates	2014-2019 Initiation, 2007-2013 Cessation	2007-2013 Initiation, 2014-2019 Cessation
2013	17.71%	17.71%	17.71%	17.71%
2014	17.17%	16.85%	17.05%	16.98%
2015	16.66%	16.05%	16.42%	16.28%
2016	16.17%	15.29%	15.83%	15.63%
2017	15.71%	14.58%	15.26%	15.02%
2018	15.27%	13.91%	14.72%	14.44%
2019	14.85%	13.28%	14.21%	13.90%

Figure A2. Estimated Smoking Prevalence from 2014-2019 under Different Values of Initiation and Cessation Rates

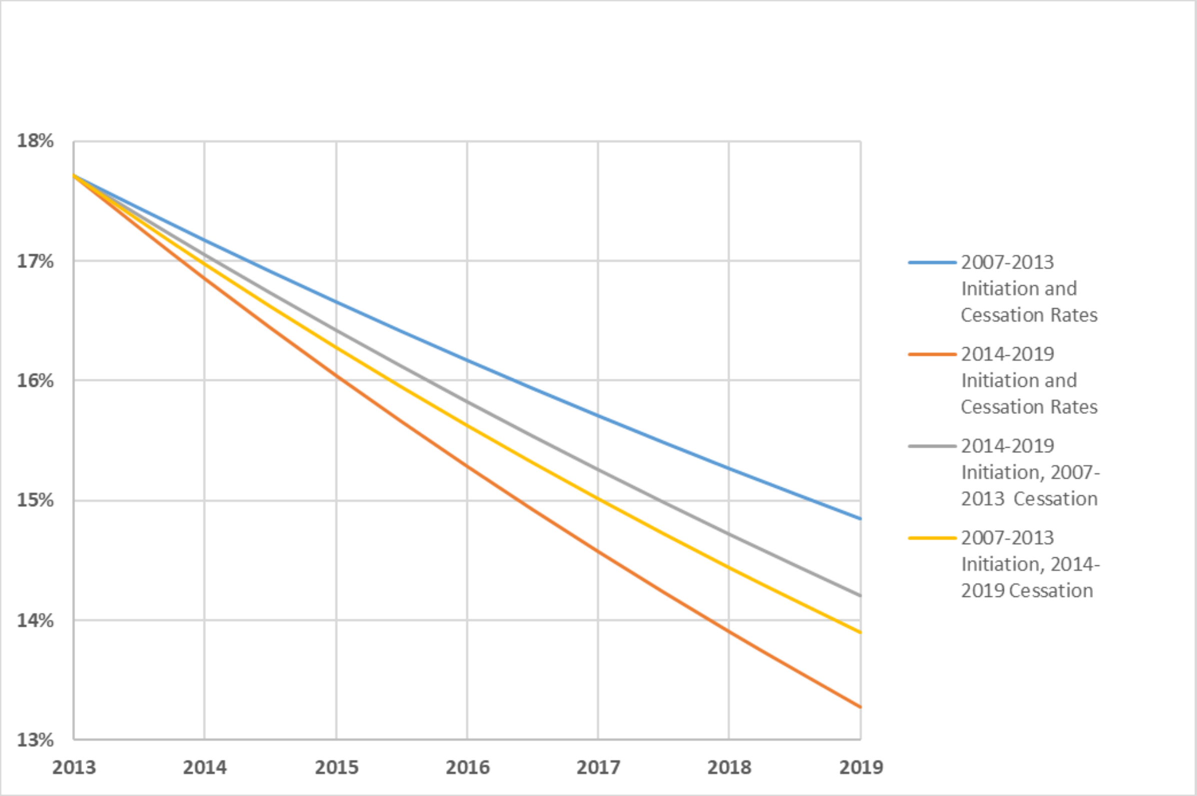

Figure A3. Drop in Smoking Prevalence Beyond Expectations from 2014-2019 Due to Recent Changes in the Initiation and Cessation Rates (2014-2019)

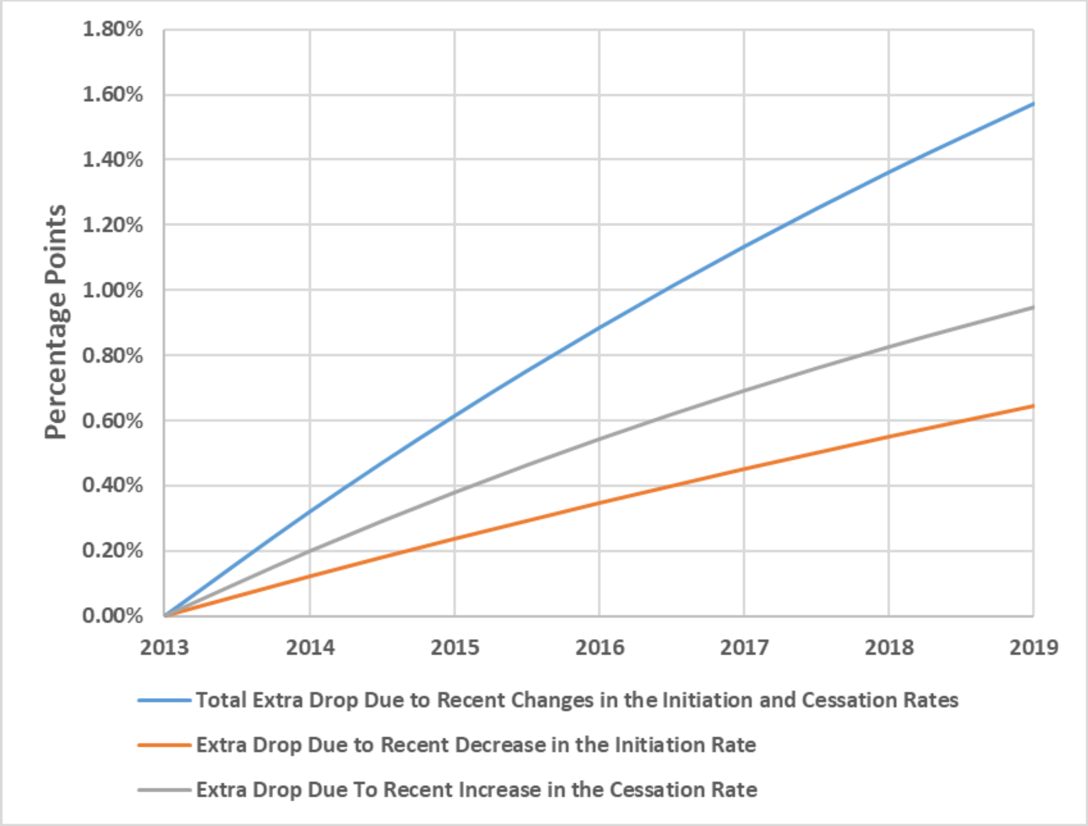

Figure A4. Relative Contributions of Recent Changes in Initiation and Cessation Rates to the Fall in Smoking Prevalence Beyond Expectation during 2014-2019

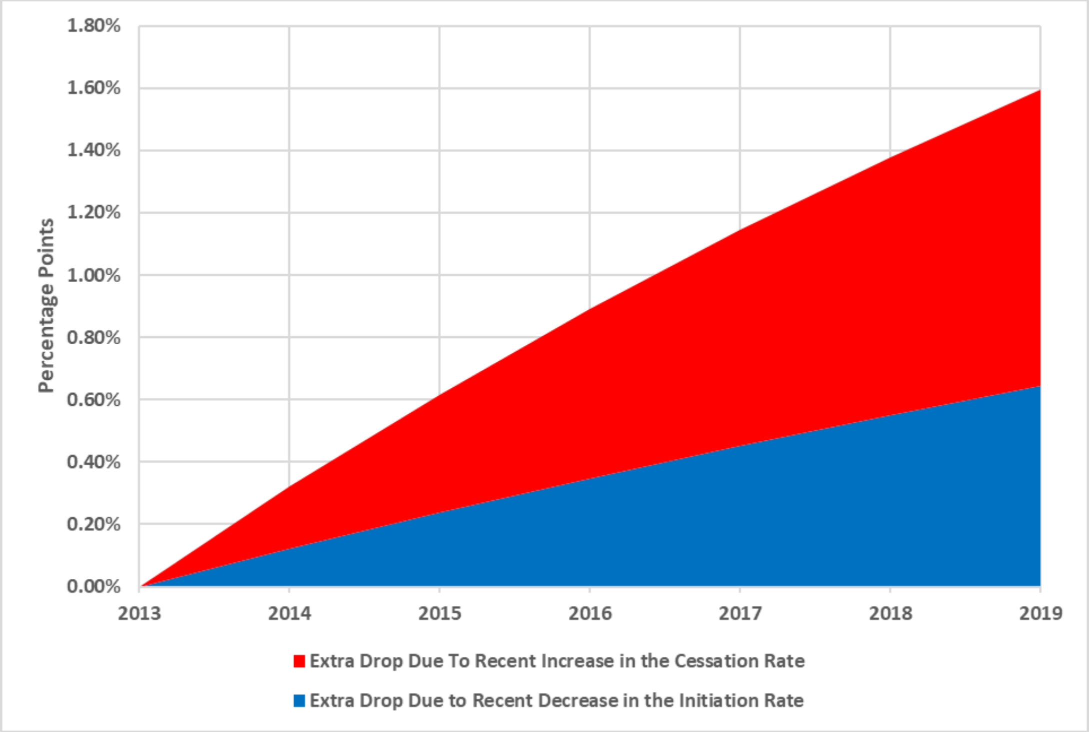

1. Méndez D, Tam J, Giovino GA, *et al.* Has smoking cessation increased? An examination of the US adult smoking cessation rate 1990–2014. *N&TR* 2017, 19(12):1418-1424.
2. Mendez D, Warner KE, Courant PN. Has smoking cessation ceased? Expected trends in the prevalence of smoking in the United States. *Am J Epidemiol* 1998, 148(3):249-258.
3. Méndez D, Alshanqeety O, Warner KE. The potential impact of smoking control policies on future global smoking trends. *Tob. control* 2013, 22(1):46-51.
4. U.S. Department of Health and Human Services. The health consequences of smoking - 50 years of progress: a report of the Surgeon General. In. Atlanta, GA: U.S. Department of Health and Human Services, Centers for Disease Control and Prevention, Office on Smoking and Health; 2014.
